# Structural Analysis of Peptide-Analogues of Human *Zona Pellucida* ZP1 Protein with Amyloidogenic Properties: Insights into Mammalian *Zona Pellucida* Formation

**DOI:** 10.1371/journal.pone.0073258

**Published:** 2013-09-12

**Authors:** Nikolaos N. Louros, Vassiliki A. Iconomidou, Polina Giannelou, Stavros J. Hamodrakas

**Affiliations:** Department of Cell Biology and Biophysics, Faculty of Biology, University of Athens, Panepistimiopolis, Athens, Greece; University of Maryland School of Medicine, United States of America

## Abstract

*Zona pellucida* (ZP) is an extracellular matrix surrounding and protecting mammalian and fish oocytes, which is responsible for sperm binding. Mammalian ZP consists of three to four glycoproteins, called ZP1, ZP2, ZP3, ZP4. These proteins polymerize into long interconnected filaments, through a common structural unit, known as the ZP domain, which consists of two domains, ZP-N and ZP-C. ZP is related in function to silkmoth chorion and in an evolutionary fashion to the teleostean fish chorion, also fibrous structures protecting the oocyte and embryo, that both have been proven to be functional amyloids. Two peptides were predicted as ‘aggregation-prone’ by our prediction tool, AMYLPRED, from the sequence of the human ZP1-N domain. Here, we present results from transmission electron microscopy, X-ray diffraction, Congo red staining and attenuated total reflectance Fourier-transform infrared spectroscopy (ATR FT-IR), of two synthetic peptide-analogues of these predicted ‘aggregation-prone’ parts of the human ZP1-N domain, that we consider crucial for ZP protein polymerization, showing that they both self-assemble into amyloid-like fibrils. Based on our experimental data, we propose that human ZP (hZP) might be considered as a novel, putative, natural protective amyloid, in close analogy to silkmoth and teleostean fish chorions. Experiments are in progress to verify this proposal. We also attempt to provide insights into ZP formation, proposing a possible model for hZP1-N domain polymerization.

## Introduction

A characteristic extracellular matrix that encloses mammalian oocytes, regulates recognition and species-specific binding of sperm and blocks polyspermy during fertilization [1]–[3]. This structure, termed *zona pellucida* (ZP), retains a protective role until embryo implantation in the endometrium has occurred [4]–[6]. It comprises glycoproteins ZP1, ZP2, ZP3 and ZP4, which are incorporated into long filaments [7]–[10]. The presence of the fourth protein, named ZP4, with structural and functional similarities to ZP1, has been associated with several, but not all mammalian species like human, rat, hamster and rabbit ZP [11]–[15]. However, protective egg coats in other vertebrates, such as the amphibians and the bird viteline envelope or fish chorion (vitelline envelope or *zona pellucida*), consist of 3 to 6 glycoproteins [16], [17]. Experimental data initially indicated that ZP filaments are formed exclusively by ZP3-ZP2 (∼90% ZP total mass) homodimers and are crosslinked through ZP1 (∼10% ZP total mass) homodimers [7], [18]. However, recent evidence suggests that ZP filaments are composed by ZP3-ZP2 and also ZP3-ZP1 dimers, which are crosslinked through ZP1 units [19]–[23].

ZP proteins undergo a distinct conformational change, crucial for secretion and polymerization, before arrangement into the filamentous matrix is possible [24]–[26]. They belong to a certain family of proteins, with a variety of functions and structures, designated as *zona pellucida* domain proteins. The only apparent similarity of this diverse group of proteins is the presence of a structural domain, about 260 amino acids long, next to their C-terminal end. This structural component, named ZP domain, exhibits a conserved intramolecular disulfide bond pattern, utterly important for its structural integrity and function [24], [27], [28]. A protease sensitive linker splits this binary structure into two functional domains, named ZP-N and ZP-C. The latter is associated with protein function and considered as a subunit interaction mediator, whereas the ZP-N domain is responsible for ZP protein polymerization [25], [29], [30].

Recent, impressive experimental data indicate that the ZP-N domain adopts an immunoglobulin-like fold [31]. It consists of two β-sheets, with strands A-B-E and C-F-G that form a β-sandwich, isolating in that manner a hydrophobic core. The presence of an extra β-strand (E′), which contributes in the formation of dimers, differentiates this structure from the classical immunoglobulin fold [32].

Amyloids are insoluble fibrous deposits, formed by misfolded protein or peptide segments, in pathological cases [33], [34]. A growing number of ‘conformational diseases’, called amyloidoses, have been associated with the formation of amyloids [35], [36]. However, occasionally, organisms exhibit novel, important biological functions, based on the functional architectures of amyloids [37]–[40]. Silkmoth chorion, which belongs to a group of protective coats surrounding oocytes from several organisms, including mammalian *zona pellucida* [4], [27], is the first well-documented case of a functional, protective amyloid [37], [41], [42]. Therefore, it is possible that the related to silkmoth chorion mammalian *zona pellucida* also retains the properties of a functional protective amyloid. We should emphasize at this point that proteins which constitute functional amyloids retain their native, properly folded, structure.

Several studies have shown that the existence of short sequence stretches with high aggregation propensity is associated with amyloid formation [43]–[46]. Sequence analysis of ZP proteins with AMYLPRED [43], a consensus aggregation propensity prediction algorithm, developed in our lab, freely available to academic users at: http://biophysics.biol.uoa.gr/AMYLPRED, revealed the presence of several “aggregation-prone” segments within human ZP1 protein sequences. Two such peptide-analogues, coinciding with the A and G β-strands of the ZP-N domain of human ZP1 (named ZPH_A and ZPH_G respectively) were predicted to have high aggregation potential and were synthesized.

In this work, we report the amyloidogenic properties of both peptides and we attempt to provide further insights into *zona pellucida* filament formation, based on our findings, suggesting that ZP proteins polymerize via their ZP-N domains, by specific interactions between such “aggregation-prone” peptides.

## Materials and Methods

### Human ZP1 ZP-N domain homology modelling

**Figure 1:**
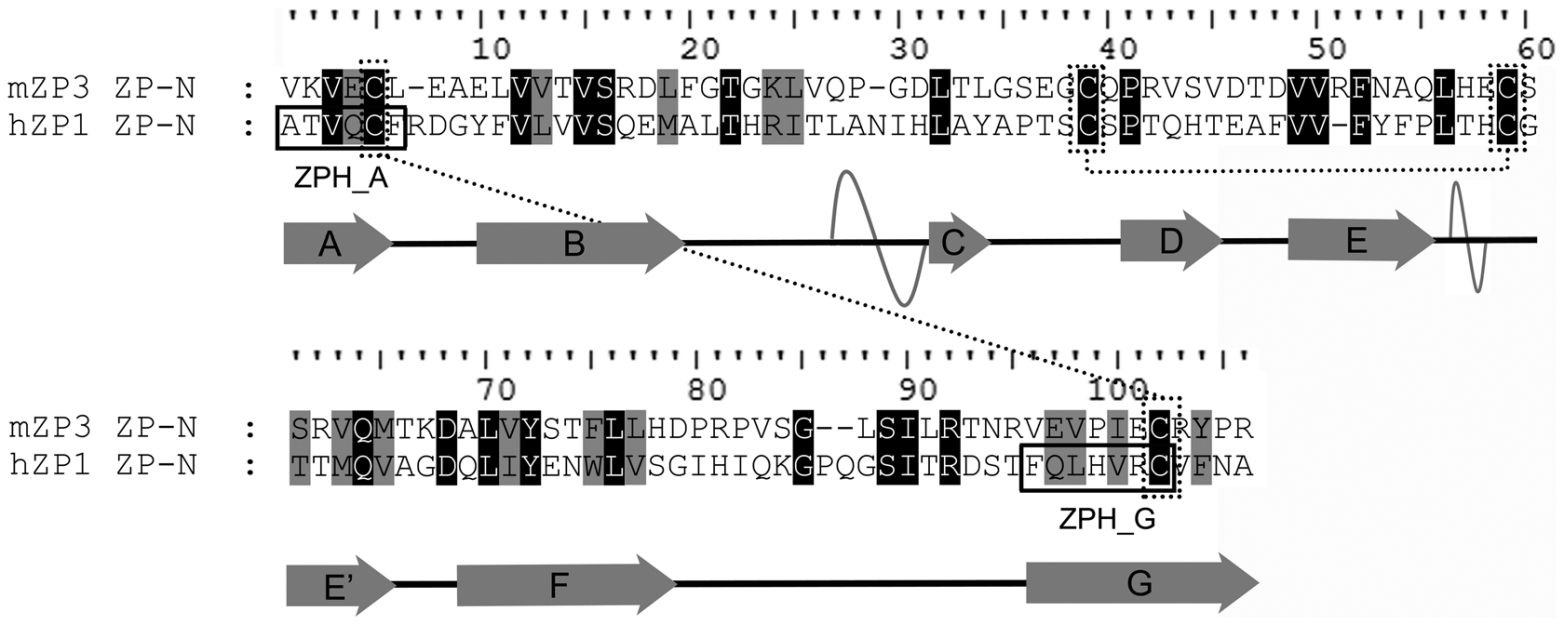
Sequence alignment of the ZP-N domain of mouse ZP3 and human ZP1 proteins. The crystallographically determined secondary structure elements are depicted below the sequences: Arrows and helices represent observed beta-strands (named consecutively A to G) and alpha helices, respectively. The invariant cysteine residues connected by disulfide bonds (dotted lines) are also seen. Peptides ATVQCF and FQLHVRC, corresponding to the beta strands A and G of human ZP1, respectively, predicted by AMYLPRED [43] as ‘aggregation-prone’ stretches, are enclosed in boxes (see ‘Materials and methods’).

Sequences of human ZP1 (hZP1) and mouse ZP3 (mZP3) proteins (**Uniprot Database Accession Numbers: P60852 and P10761**, respectively), were extracted from Uniprot [47]. Sequence alignment was performed using ClustalW [48], [49]. Subsequently, the sequence fragment corresponding to the ZP-N domain of hZP1 was derived and isolated based on this alignment (Fig. 1). Finally, a model of the three-dimensional structure of the hZP1 ZP-N domain was derived, based on this alignment, utilizing MODELLER 9v2 [50], [51].

### Prediction of potential “aggregation-prone” segments and peptide synthesis

The hZP1 ZP-N domain sequence was then processed, utilizing our consensus prediction algorithm, “AMYLPRED”, and potential “aggregation-prone” peptides were predicted.

Two “aggregation-prone” predicted peptides, from the ZP-N domain of hZP1, were synthesized (amino acid residues 276–281 and 370–376, respectively: numbering that of P60852), matching the A strand (ATVQCF) and the G strand (FQLHVRC) of the modelled hZP1 ZP-N domain, called ZPH_A and ZPH_G correspondingly, with a simple substitution of the Cysteine (C) residues by Alanines (A), respectively, to avoid formation of undesired disulfide bonds, at neutral pH. Thus, the two synthesized peptides were: ZPH_A, ATVQAF and ZPH_G, FQLHVRA, respectively. Peptides were synthesized by GeneCust Europe, Luxembourg (purity >98%, free N- and C-terminals). Both synthesized peptides were dissolved in distilled water (pH 5.5, concentration 10 mg ml^−1^) and were incubated for a time period of 1–2 weeks. The two peptides were found to fold and self-assemble into amyloid-like fibrils, producing gels. Mature amyloid-like fibrils are formed after an inclubation period of ca. 1–2 weeks, at ambient temperatures.

### Negative staining and transmission electron microspopy (T.E.M.)

The two synthesized peptides were, independently to each other, dissolved in distilled water (pH 5.5) at a concentration of 10 mg ml^−1^, each. They were both found to form amyloid-like fibrils after an incubation period of ca. one week, at ambient temperatures. Similarly, a solution of a mixture of the ZPH_A and ZPH_G peptides (in 1∶1 ratio, in distilled water, pH 5.5, concentration 5 mg ml^−1^ per peptide) also revealed the formation of amyloid-like fibrils, after an incubation period of ca. one week. We should like to note here that the mixing of the two peptides ZPH_A and ZPH_G (lyophilized powders) was performed before dissolving them into distilled water.

Fibril suspensions, were applied to glow-discharged 400-mesh carbon and plastic-coated copper grids for 60 s, after an incubation period of ca. 2 weeks, so that the fibrils were mature (no changes in appearance and characteristics). The grids were stained with a drop of 1% (w/v) aqueous uranyl acetate for 45 s. Removal of excess stain was performed in air, by blotting with a filter paper. The grids were air-dried and examined in a Philips CM120 BioTWIN electron microscope (FEI, Eindhoven, The Netherlands) operated at 100 kV. Digital acquisitions where performed with a bottom-mounted Keen View 1K CCD camera (Soft Imaging System, Muenster, Germany). Grids containing fibrils from the ZPH_A and ZPH_G mixture, were examined with a Morgagni™ 268 transmission electron microscope, operated at 80 kV. Digital acquisitions were perfomed with an 11 Mpixel side-mounted Morada CCD camera (Soft Imaging System, Muenster, Germany).

### Congo red staining and polarized light stereomicroscopy

Fibril suspensions of all three peptide solutions were applied to glass slides and were allowed to air-dry. The film formed by the ZPH_A peptide was stained with a 10 mM Congo red solution in phosphate-buffered saline (pH 7.4) for approximately 1 h [52]. Excess staining was removed by several washes with 90% ethanol and left to dry. The film produced by the ZPH_G peptide was stained with a 1% Congo red solution in distilled water (pH 5.75) at room temperature for approximately 20 minutes, as indicated by the standard Rományi protocol [53]. Excess stain was removed through tap water washes as also indicated by the same protocol [53], [54]. Subsequently, the samples were observed under bright field illumination and between crossed polars, using a Leica MZ75 polarizing stereomicroscope equipped with a JVC GC-X3E camera.

### X-ray diffraction

The peptides were dissolved in distilled water (pH 5.5) at a concentration of 15 mg ml^−1^ to produce amyloid-like fibrils after 1 week incubation. A peptide mixture containing both peptides in equal ratios (1∶1) was also dissolved in distilled water (pH 5.5, see ‘Negative staining’ above). In order to obtain oriented fibers, suitable for X-ray diffraction, a (∼10 µl) droplet of fibril suspension was placed between two siliconized glass rods, spaced ∼2 mm apart and mounted horizontally on a glass substrate, as collinearly as possible. The droplet was allowed to dry slowly at ambient temperature and humidity for approximately 1 h to form an oriented fiber appropriate for X-ray diffraction. X-ray diffraction patterns were recorded on a Mar Research 345 mm image plate, utilizing CuK_α_ radiation (λ = 1.5418 Å), obtained from a Rigaku MicroMax-007 HF, microfocus rotating anode generator (with Osmic Rigaku VariMax™ HF optics), operated at 50 kV, 100 mA. The specimen-to-film distance was set at 150 mm and the exposure time was 30 minutes. No additional low angle reflections were observed at longer specimen-to-film distances of up to 300 mm. The X-ray patterns, initially viewed using the program MarView (MAR Research, Hamburg, Germany), were displayed and measured with the aid of the program IPDISP of the CCP4 package [55]. X-ray diffraction patterns produced by fibers of the mixed fibril suspension of ZPH_A and ZPH_G peptides were collected, using a SuperNova-Agilent Technologies X-ray generator equipped with a 135-mm ATLAS CCD detector and a 4-circle kappa goniometer, at the Institute of Biology, Medicinal Chemistry and Biotechnology, National Hellenic Research Foundation (CuK_α_ high intensity X-ray micro-focus source, λ = 1.5418 Å), operated at 50 kV, 0.8 mA. The specimen-to-film distance was set at 52 mm and the exposure time was set to 10 min. The X-ray patterns, initially viewed using the program CrysAlisPro [56] where displayed and measured with the aid of the program iMosFLM [57].

### Attenuated total reflectance Fourier-Transform Infrared (ATR FT-IR) spectroscopy and post-run spectra computations

Five-microliter drops of all peptide fibril suspensions (obtained from solutions, 5 mg ml^-1^, in distilled H_2_O, pH 5.5), were cast on flat stainless-steel plates coated with an ultra thin hydrophobic layer (SpectRIM, Tienta Sciences, Inc. Indianapolis, USA) and left to air-dry slowly at ambient conditions to form hydrated thin films. IR spectra were obtained at a resolution of 4 cm^−1^, utilizing an IR microscope (IRScope II, BrukerOPTICS, Bruker Optik GmbH, Ettlingen, Germany), equipped with a Ge ATR objective lens (20×) and attached to a FT-IR spectrometer (Equinox 55, BrukerOPTICS). Ten 32-scan spectra were collected from each sample and averaged to improve the S/N ratio. All spectra are shown in the absorption mode after correction for the wavelength-dependence of the penetration depth (d_p_ analogous λ). Derivatives were computed analytically using routines of the Bruker OPUS/OS2 software including smoothing over a +/− 8 cm^−1^ range around each data point, performed by the Savitsky–Golay algorithm [58]. Smoothing over narrower ranges resulted in deterioration of the signal to noise ratio and did not increase the number of minima that could be determined with confidence. The minima in the second derivative were used to determine the corresponding absorbtion band maxima.

### Docking

The derived model of the ZP-N domain of hZP1 was used to perform docking experiments, in order to evaluate the position of the predicted “aggregation-prone” peptides as a potential interface for ZP protein polymerization. Unfortunately, no further experiments could be performed in terms of the full length ZP domain, since there is no available structure for the intact polymerizing ZP domain (the full length structure of the ZP domain of chicken ZP3 was not taken into account, since it represents the form of the ZP domain that, as a precursor, inhibits ZP protein polymerization [30]). For deriving a structural model of a ZP1-N homodimer, the HADDOCK version 2.1 [59] was used using the web server version [59]. HADDOCK is a modeling approach that may incorporate structural knowledge of the target to drive the docking procedure, the latter derived from various experimental or computational methods [60]. CNS1.2 was utilized for performing the structure calculations [61]. Non-bonded interactions were calculated with the OPLS force field [62] using a cutoff of 8.5 Å. The electrostatic potential (E_elec_) was calculated by using a shift function, while a switching function (between 6.5 and 8.5 Å) was used to define the van der Waals potential (E_vdw_). The HADDOCK score is used to rank the generated poses, being the weighted sum of intermolecular electrostatic (E_Elec_), van der Waals (E_vdW_), desolvation (ΔG_solv_) and ambiguous interaction restraint (AIR) energies with weight factors of 0.2, 1.0, 1.0 and 0.1, respectively. The solvated docking protocol [63] was preferred which explicitly accounts for solvent during the docking procedure [64] since in comparison to unsolvated docking it may yield higher quality docking predictions [65]. Interaction restraints to drive the docking were set unambiguously (are not subjected to random removal) and were defined as follows. Ten pairs of C_a_ interatomic distances of 5±1 Å were set (concerning residues 277–280 and 371–375) to satisfy the modeling of a ZP1-N homodimer where β-strands A and G are interacting with their respective counterparts in an antiparallel spatial arrangement (a parallel spatial arrangement is not feasible).

## Results

**Figure 2:**
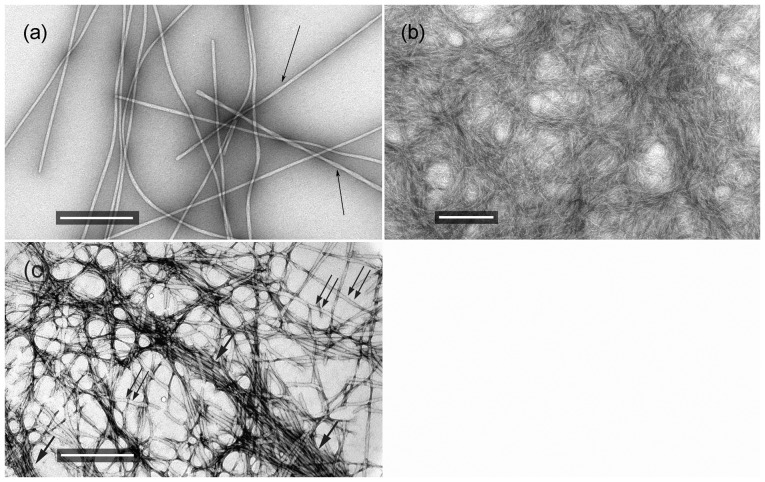
Electron micrographs of amyloid-like fibrils, negatively stained with 1% uranyl acetate. Amyloid-like fibrils were derived by self-assembly, from a 10 mg ml^−1^ solution of peptides ZPH_A (a) and ZPH_G (b) in distilled water, pH 5.5. A solution of a mixture of the ZPH_A and ZPH_G peptides (in 1∶1 ratio, in distilled water, pH 5.5, concentration 5 mg ml^−1^ per peptide) also revealed the formation of amyloid-like fibrils, after an incubation period of ca. one week (c). (a) They are unbranched and of undetermined length, approximately 100–120 Å in diameter and have a double helical structure. A pair of protofilaments each 40–50 Å in diameter wrap around each other, with intervening stain between them, thus forming double-helical fibrils (arrows). Bar 500 nm. (b) Protofilaments interact laterally, forming ribbons and, eventually, gels. The fibrils formed exhibit a characteristic for amyloid-like fibrils polymorphism [67]–[69]. Bar 200 nm. (c) Two different types (populations) of fibrils are apparent, due to “self-aggregation” of each peptide (double and single arrows, respectively), which are similar to those viewed separately by the ZPH_A and ZPH_G peptide solutions in (a) and (b) above. Bar 500 nm.

Both peptides predicted by our algorithm, AMYLPRED, as possible “aggregation-prone” regions of hZP1-N, were examined thoroughly, after synthesis and were found to self-assemble into amyloid-like fibrils, forming gels, after incubation for 1–2 weeks, in distilled water (pH 5.5). Specifically, TEM and negative staining revealed that fibrils, structurally uniform in diameter, are formed due to the self-assembly of peptide ZPH_A, with a thickness of approximately ∼100–120 Å. These fibrils are characterized as amyloid-like, since they appear to be straight and unbranched double helices, of undetermined length, which consist of protofilaments, also with a uniform diameter of approximately 40–50 Å and they exhibit all the characteristics of amyloid-like fibrils (see below and Fig. 2a) [66]. Polymerization of the ZPH_G peptide, under the same conditions leads to the formation of denser gels. Ribbons, varying in thickness, of fibrils interacting laterally are formed and, also, supercoiled fibrils with various diameters (Fig. 2b). This apparent morphological polymorphism of amyloid fibrils has been exhibited by many different amyloid-forming peptides or proteins [67]–[69]. A solution of a mixture of the ZPH_A and ZPH_G peptides also revealed the formation of fibrils, which, apparently, form two separate populations of fibrils that exhibit the properties of the fibrils produced by the individual ZPH_A and ZPH_G peptides, instead of the formation of fibrils due to interacting peptides (Fig. 2c).

**Figure 3:**
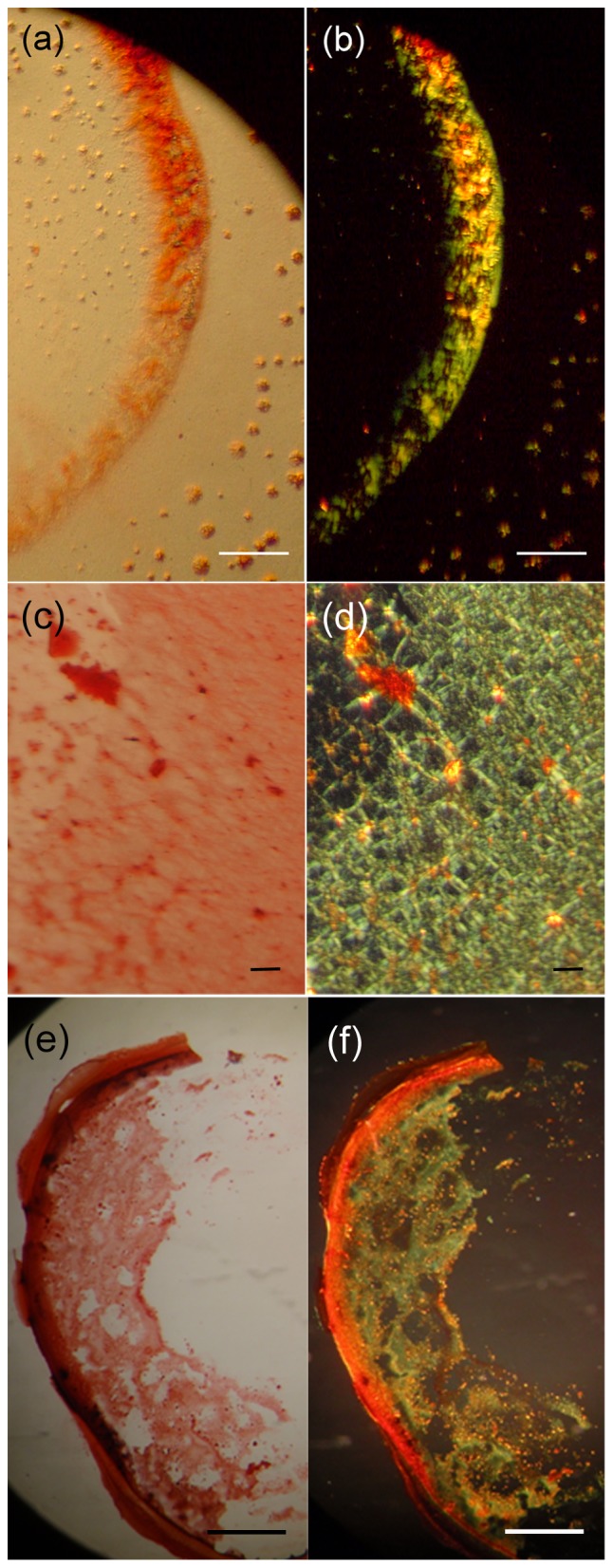
Photomicrographs of peptide fibrils stained with Congo red. Fibrils have derived from: ZPH_A (a–b), ZPH_G (c–d) and ZPH_A & ZPH_G mixture (e–f) peptides, respectively (see ‘Materials and methods’). Solutions of these peptides, after ca. one (1) week incubation, produced fibrils, which were then stained with Congo red. The typical for amyloid fibrils apple-green birefringence is clearly seen, under crossed polars. (a,c,e) Bright field illumination, (b,d,f) Crossed polars Bar 400 µm.

It is well established that amyloid-like fibrils bind Congo red [52] and therefore gels produced by these peptides were stained and examined under a polarizing microscope. In all three cases, amyloid deposits bind Congo red, as it is clear under bright field illumination and exhibit a characteristic for amyloid fibrils apple/green birefringence, when viewed under crossed polars (Fig. 3).

**Figure 4:**
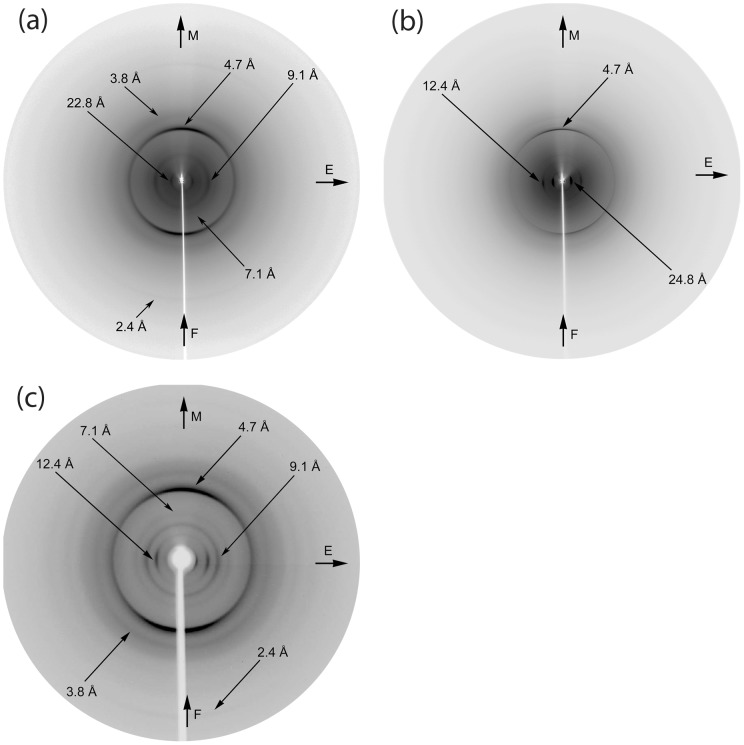
X-ray diffraction patterns produced from oriented fibres of mature fibril suspensions. The mature fibrils have derived from: (a) ZPH_A peptide, (b) ZPH_G peptide, (c) a mixture of ZPH_A & ZPH_G peptides. The meridian, M (direction parallel to the fibre axis, F) is vertical and the equator, E, is horizontal in this display. All X-ray diffraction patterns are clearly “cross-β” patterns [71]–[73]. (a) An intense meridional 4.7 Å reflection corresponds to the spacing of successive hydrogen bonded β-strands, perpendicular to the fiber axis, whereas the 9.1 Å reflection on the equator is attributed to the packing distance of β-sheets (Table 1, column 4). The sheets are packed parallel to the fiber axis. (b) The X-ray diffraction pattern of the ZPH_G peptide also exhibits similar reflections that indicate the presence of a “cross-β” conformation. The structural repeat of 4.7 Å corresponds to the spacing of successive β-strands arranged perpendicular to the fiber axis, while the 12.4 Å spacing on the equator, corresponds to the packing distance of consecutive β-sheet parallel to the fibre axis (Table 1, column 5). (c) The X-ray pattern produced from the mixture of the ZPH_A & ZPH_G peptide fibril suspensions is clearly a combination of the diffraction patterns produced by the individual fibers formed from the ZPH_A and ZPH_G peptide's fibril suspensions (Table 1, column 6). The 2.4 Å, 3.8 Å, 7.1 Å and 9.1 Å reflections are due to the presence of fibrils formed by the ZPH_A peptide, whereas the 12.4 Å reflection is produced by fibrils formed by the ZPH_G peptide (corresponding to β-sheet packing distance). The intense 4.7 Å reflection has contributions from both fibril populations and this is in agreement with the EM photograph of Fig. 2c.

Aligned fibers, produced by more or less oriented fibrils from the above solutions, present X-ray diffraction patterns with the clear characteristics of a “cross-β” conformation, observed for most amyloids (Table 1) [70]–[73]. The long axis of the oriented amyloid-like fibrils is relatively parallel to the fiber axis. The X-ray pattern produced from oriented fibers of fibrils formed by the ZPH_A peptide, reveals an intense reflection on the meridian (perpendicular to the fiber axis) corresponding to a periodicity of 4.7 Å. This periodicity refers to the distance between consecutive hydrogen bonded β-strands, aligned perpendicular to the long axis of the fibrils and therefore also to the axis of the oriented fiber. The reflection on the equator, corresponding to a repeat of 9.1 Å, is attributed to the packing distance between successive packed β-sheets, parallel to the fiber axis (Fig. 4a). The X-ray pattern produced from the ZPH_G peptide ‘oriented’ fibers, is also indicative of a “cross-β” conformation, displaying a 4.7 Å reflection on the meridian, corresponding to the distance between successive hydrogen bonded β-strands and a 12.4 Å reflection on the equator, corresponding to the distance between packed β-sheets (Fig. 4b). The inclusion of larger side chains like those of Arg (R) in this peptide, possibly explains the larger packing distance between ‘packed’ beta-sheets. A reflection at 24.8 Å (Fig. 4b), may be the second order of the 12.4 Å reflection or may perhaps be attributed to tandem repeats of the length of this peptide (24.8 Å = 7 residues×3.5–3.6 Å per residue), arising from the interaction of adjacent molecules.

**Table 1:** Spacings of the reflections observed in the X-ray diffraction patterns taken from oriented fibers of ZPH_A and ZPH_G peptides-derived amyloid fibrils, and from an oriented fiber, produced from self-assembled fibrils, formed from a mixture of both ZPH_A and ZPH_G peptides, dissolved in equal ratios (1∶1) (see ‘Materials and methods’ and Fig. 4).

h	k	l	d_obs_ (Å)		
			ZPH_A	ZPH_G	ZPH_A & ZPH_G
0	2	0		24.8	
1	0	0	22.8		
0	1	0	9.1	12.4	9.1 & 12.4
2	1	0	7.1		7.1
0	0	1	4.7	4.7	4.7
6	0	0	3.8		3.8
3	3	1	2.4		2.4

Solutions containing both peptides, dissolved in equal amounts (1∶1), formed fibers from oriented fibrils, that produced an X-ray pattern that appears to be a combination of the patterns produced by each peptide separately. Specifically, the 2.4 Å, 3.8 Å, 7.1 Å and 9.1 Å reflections, correspond to the pattern produced by ZPH_A fibers, while the 12.4 Å periodicity, is produced by ZPH_G fibers. We should mention that the meridional 4.7 Å reflection, apparently, arises from the contribution of both peptides forming amyloid-like fibrils (Fig. 4c).

**Figure 5:**
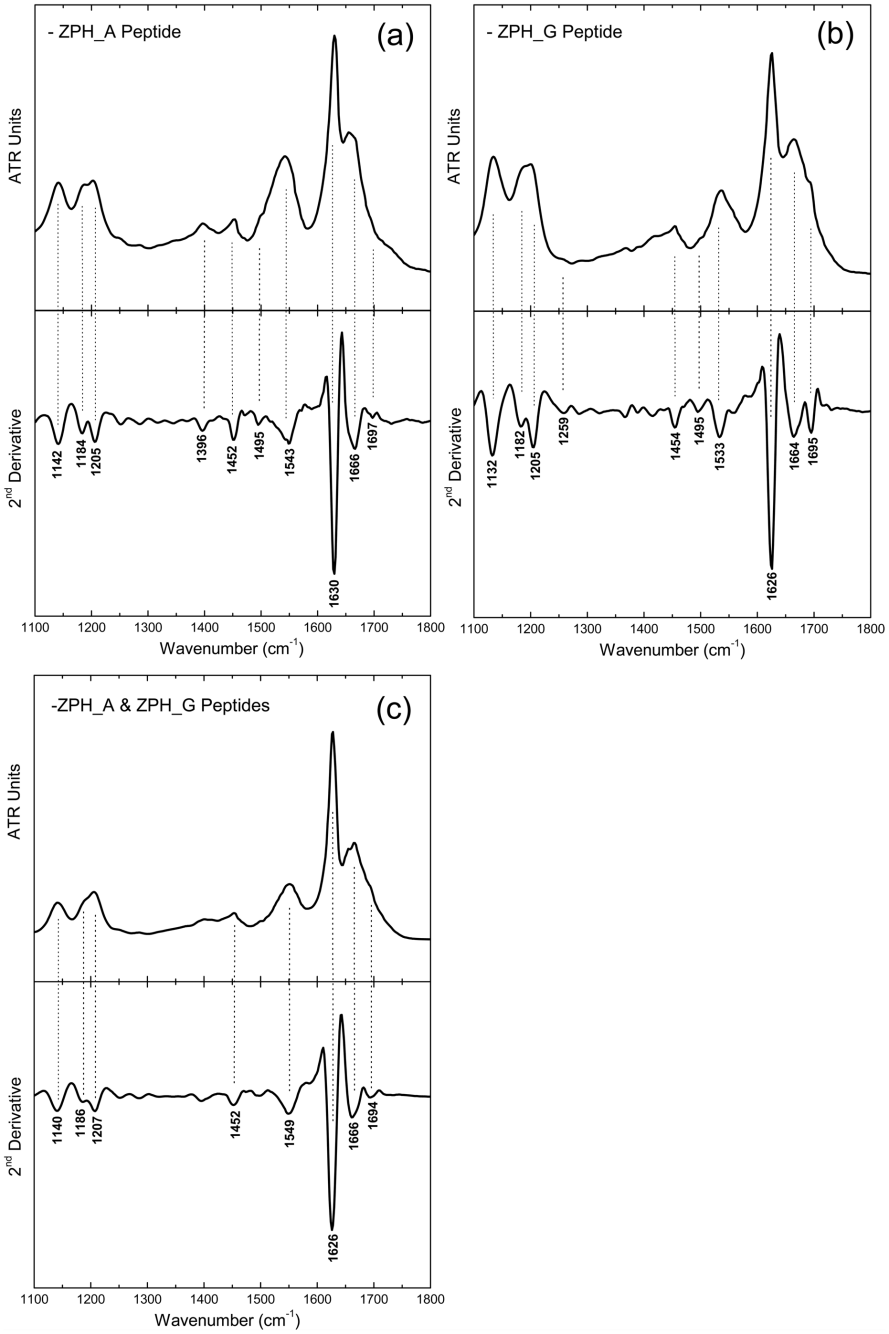
ATR FT-IR (1100–1800 cm^−1^) spectra obtained from thin hydrated-films containing mature amyloid-like fibrils. These thin hydrated-films, formed from (a) ZPH_A, (b) ZPH_G and (c) the mixture of ZPH_A & ZPH_G peptides, were cast on flat stainless-steel plates coated with an ultra thin hydrophobic layer (see ‘Materials and methods’). Second derivative spectra are also included and were used for the exact identification of the band maxima and their tentative assignments. All resulting spectra are indicative of the preponderance of an antiparallel β-sheet secondary structure (Table 2).

Spectral acquisition by ATR FT-IR spectroscopy has been shown to yield rich information about the secondary structure of peptides forming amyloid fibrils, without the drawbacks associated with the more conventional vibrational techniques[37]. Therefore, ATR FT-IR spectra were obtained from both peptide solutions and their mixture, when mature amyloid fibrils had been formed. More specifically, the ATR FT-IR spectrum produced from ZPH_A fibril-containing suspensions, cast as hydrated-thin films on a suitable substrate (see Methods), shows an Amide I band at 1630 cm^−1^ indicative of the preponderance of β-sheet conformation [74]–[78], supported by an additional band at 1697 cm^−1^, most probably due to the presence of antiparallel β-sheets (Fig. 5a) [75]–[77]. Similarly, the spectrum produced from ZPH_G peptide thin films reveals bands at 1626 cm^−1^ and 1695 cm^−1^ (Fig. 5b). Finally, the spectrum produced from thin films formed by a solution of peptides ZPH_A and ZPH_G (in a 1∶1 ratio) containing mature fibrils, also revealed similar bands at 1627 cm^−1^ and 1695 cm^−1^ (Fig. 5c). Conclusively, in all cases, the resulting ATR FT-IR spectra strongly support the presence of an antiparallel β-sheet conformation (Table 2).

**Table 2:** Bands observed in the IR spectrum of a hydrated film produced from a suspension of fibrils produced by ZPH_A peptide, ZPH_G peptide and from a mixture of both peptides, dissolved in equal (1∶1) amounts and their tentative assignments (Fig. 5).

Bands (cm^−1^)			Assignments
ZPH_A	ZPH_G	ZPH_A & ZPH_G	
1142	1132	1141	TFA
1184	1182	1186	TFA
1205	1205	1205	TFA
1396			CH_3_ Bending
1452	1454	1454	CH_2_ Deformation
1495	1495		Phe
1543	1533	1550	β-sheet (Amide II)
1630	1626	1627	β-sheet (Amide Ι)
1666	1664	1666	TFA
1697	1695	1695	Antiparallel β-sheet (Amide I)

## Discussion

**Figure 6:**
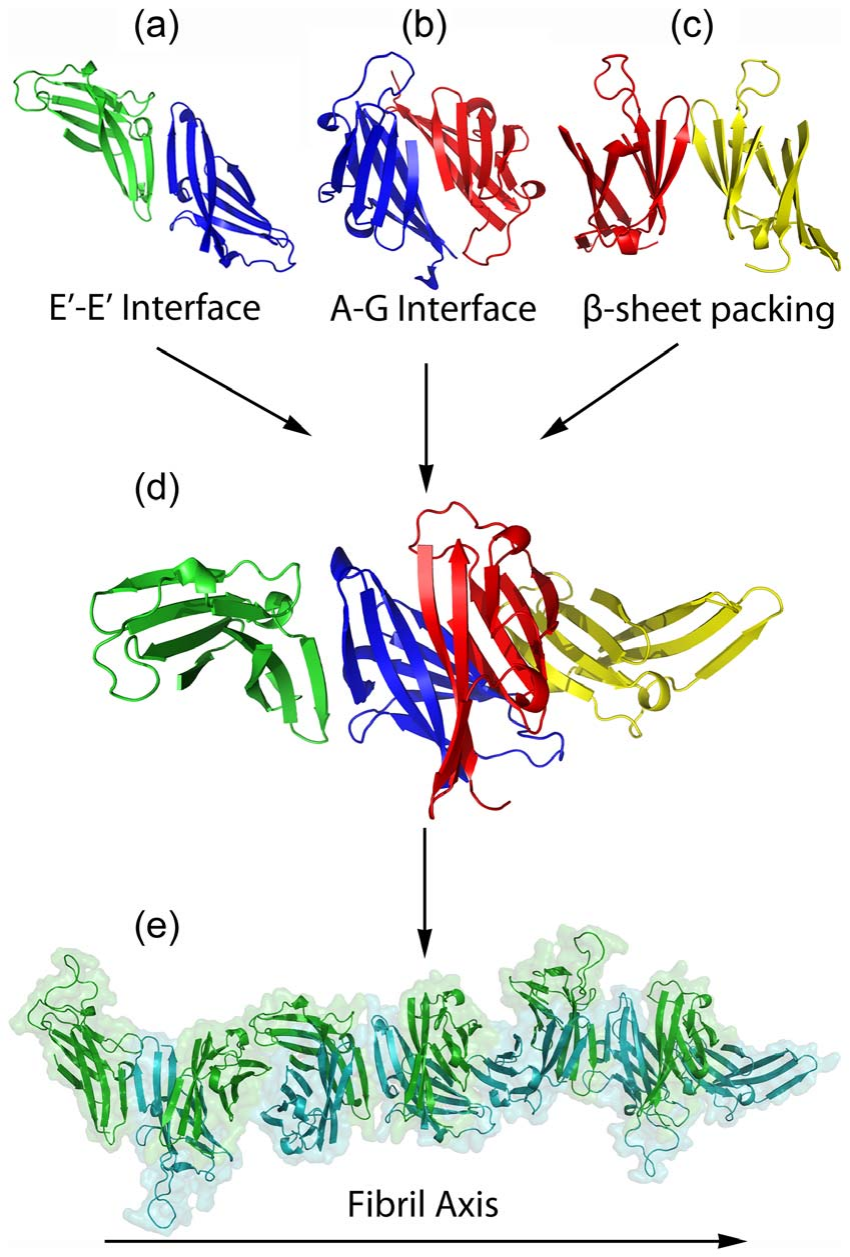
Schematic representation of the ZP-N domain polymerization process. (a) A structural model exhibiting the formation of ZP-N dimers due to interactions, between E′-E′ β-strands [32]. These are real crystal contacts [32]. The two ZP-N units are shown in green and blue, respectively. (b) ZP-N dimers formed by our docking attempts, utilizing the software HADDOCK [59], [60], which led to the identification of a possible ‘AG interface’. The A and A β-strands of each monomer interact favorably, to form a dimer, and, this is also true for the corresponding G and G strands of each monomer. These favorable interactions are clearly seen in the current experimental work. The two ZP-N units are shown in blue and red, respectively. (c) ZP-N dimers are also formed by close packing of their adjacent β-sheets, as it has been previously shown, in the crystal structure of the ZP-N domain of ZP3 [32]. The two ZP-N monomers are colored in red and yellow, respectively. (d) A combination of all three possible interactions between successive ZP-N monomers, results in the formation of a specific ZP-N domain tetramer. ZP-N monomers at each side retain a free ‘AG interface’ that allows for successive tetramers to further polymerize. Each monomer is shown in different colour. (e) Cartoon and surface representation of the polymerization process of the ZP-N domain. Consecutive tetramers are brought together by favorable interactions between their ‘AG interfaces’, resulting in the formation of a linear, helical β-strucure, representing the main fibril axis of a ZP protofilament. The diameter of this proposed model is approximately 60 Å. This structure is composed of 18 ZP-N blocks (shown in cyan and green) interacting through alternating AG, E′-E′ interfaces and β-sheet close packing.

As mentioned in the Introduction, it has been shown experimentally that, the ZP-N domain adopts an immunoglobulin-like fold, with an extra β-strand (E′), differentiating this fold from the classical immunoglobulin fold and contributing to the formation of ZP-N dimers [32] (Fig. 6A).

All experimental data corroborate our prediction, based on the AMYLPRED results, that ZPH_A and ZPH_G peptides are “aggregation-prone”, since they were found to self-assemble into fibrils that fulfill all the basic criteria of amyloids. Experiments with peptide solutions containing both peptides, dissolved in equal amounts (1∶1) clearly revealed that there are indeed two discrete fibril populations formed, that are identical to those formed by each peptide separately, indicating that both peptides are “self-aggregating”.

A detailed study of the modeled three-dimensional structure of the hZP1 ZP-N domain, provided information for the location of both peptides within this functional unit. As it is apparent, β-strands A and G lie facing each other, forming a potential molecular interface (Fig. 6b) (‘AG interface’). Docking procedures (see ‘Materials and methods’) indicated that ZP1 dimers may be formed due to interactions between AG interfaces of their ZP-N domains, as perhaps expected, supporting our hypothesis that these segments are “aggregation-prone” and introducing a possible lead for ZP-N polymerization. The optimal solution provided a HADDOCK score of −113.1 (Van der Waals energy of −44.9, electrostatic energy of −373.8, desolvation energy of +5.1 and restraints violation energy of +14.0 Kcal*mol^−1^, respectively) with a total buried surface area of 1682.3 Å^2^.

Our results may provide further insights into the formation of the main axis of ZP filaments, since the ‘AG interface’ has already been proposed as a potential interface for ZP protein polymerization and it is considered as a conserved and fairly exposed hydrophobic region, lying between the A and G strands of all ZP proteins [31]. Combining all our findings and the above information on the ZP-N domain structure, it is possible that ZP3-ZP1 and ZP3-ZP2 heterodimers that constitute the main axis of ZP fibrils [19]–[23], could be formed through similar interactions between their AG interfaces. However, a similar structural analysis of the related AG interfaces of the ZP-N domains of hZP2 and hZP3 proteins in combination with the work on the hZP1 AG interface, would strengthen this assumption (work in progress). Furthermore, as previously noticed, ZP-N blocks are known to form dimers, either through intermolecular backbone interactions (hydrogen bonds) between their extra E′ β-strands (E′-F-G extension) (Fig. 6a) or through their β-sheet close packing (Fig. 6c), in the crystal structure of the ZP-N domain of mZP3 [32]. These established interactions could possibly contribute in interconnecting successive ZP protein heterodimers formed through the AG interaction. To summarize, a generalized model depicting the polymerization of ZP proteins into filaments could indicate that ZP protein heterodimers (either ZP3-ZP2 or ZP3-ZP1) are formed through interactions of their “aggregation-prone” AG interfaces and these dimers are interconnected through alternating E′-E′ and β-sheet close packing interfaces (Fig. 6d). Subseqeuntly, succesive repeats of ZP3-ZP2 and ZP3-ZP1 may interact in a tandem fashion, resulting in the formation of a filamentous structure of perpetually twisted β-sheets, as an aftereffect of alternating AG, E′E′ interfaces and β-sheet close packing (Fig. 6e). The role of the ZP-C domains in the polymerization process is still not clear.

Amyloid protofilaments commonly form intertwined helical β-structures [70], [79], a notion that is in agreement with our model. Antiparallel β-strands constituting the filament backbone are ordered more or less perpendicular to its main axis, in the sense of a “cross-β” conformation.

## Conclusions

Our experimental data suggest that certain peptides of the ZP proteins, possibly have the self-assembly potential to drive ZP proteins into amyloid fibrils, since it has been previously stated that the ZP-N domain of ZP proteins alone is responsible for ZP protein polymerization [25], [29], [30]. These peptides may have a similar role in the context of the entire hZP1 protein sequence and structure, and perhaps, their equivalents similar roles to the the hZP2-4 proteins as well. This, in turn, may imply that, mammalian *zona pellucida* is another novel functional amyloid, similar to its functional equivalent, silkmoth chorion that has long been established as a natural, protective amyloid [37], [41], [42], or the teleostean fish chorion, a biological equivalent of mammalian ZP, which consists of fish ZP proteins homologous to mammalian ZP proteins and which succesfully binds the Congo red dye, exhibiting the apple-green birefringence [80]. Experiments are in progress to determine whether this is true or not in the case of the mammalian ZP. These experiments are necessary in order to establish whether mammalian ZP indeed retains amyloidogenic properties to its full extent. In essence, although the AG interface, formed by the predicted aggregation-prone ZPH_A and ZPH_G peptides, clearly possesses amyloidogenic properties, it accounts merely for a part of the ZP-N domain of mammalian ZP proteins. Consequently, our results may not be enough to establish if and how amyloid fibrils are formed by mammalian ZP proteins *in vivo*, however, they provide information about the formation of the heterodimer bulding blocks of mammalian ZP filaments.

The outcome of these experimental results could prove to be of importance, since the “aggregation-prone” segments that we have detected, are not found buried within the ZP-N domain interior core, but, instead, reside on the surface of the proteins and hence, could serve as targets for inhibition of ZP protein polymerization. Consequently, novel contraception drugs or even improved fertility methods and medication could be designed, by exploring and targeting the structural properties of these amyloidogenic “edges” of the ZP proteins, following the examples of pathological amyloid treatments [81], [82].

Finally, our findings may provide further insights that could prove to be essential in exploiting the intriguing mechanical properties of *zona pellucida* or its similar structures, since functional amyloids might serve as a source for biomaterial engineering/designing [83], [84].
